# Development of methane conversion factor models for Zebu beef cattle fed low‐quality crop residues and by‐products in tropical regions

**DOI:** 10.1002/ece3.2500

**Published:** 2016-09-26

**Authors:** Chatchai Kaewpila, Kritapon Sommart

**Affiliations:** ^1^ Department of Animal Science Faculty of Agriculture Khon Kaen University Khon Kaen Thailand

**Keywords:** cattle, low‐quality feed, meta‐analysis, methane, tropical countries, *Y*_m_ model

## Abstract

The enteric methane conversion factor (*Y*_m_) is an important country‐specific value for the provision of precise enteric methane emissions inventory reports. The objectives of this meta‐analysis were to develop and evaluate the empirical *Y*_m_ models for the national level and the farm level for tropical developing countries according to the IPCC's categorization. We used datasets derived from 18 in vivo feeding experiments from 1999 to 2015 of Zebu beef cattle breeds fed low‐quality crop residues and by‐products. We found that the observed *Y*_m_ value was 8.2% gross energy (GE) intake (~120 g methane emission head^−1^ day^−1^) and ranged from 4.8% to 13.7% GE intake. The IPCC default model (tier 2, *Y*_m_ = 6.5% ± 1.0% GE intake) underestimated the *Y*_m_ values by up to 26.1% compared with its refinement of 8.4% ± 0.4% GE intake for the national‐level estimate. Both the IPCC default model and the refined model performed worse in predicting *Y*_m_ trends at the farm level (root mean square prediction error [MSPE] = 15.1%–23.1%, concordance correlation coefficient [CCC] = 0.16–0.18, *R* ^2^ = .32). Seven of the extant *Y*_m_ models based on a linear regression approach also showed inaccurately estimated *Y*_m_ values (root MSPE = 16.2%–36.0%, CCC = 0.02–0.27, *R* ^2^ < .37). However, one of the developed models, which related to the complexity of the energy use efficiencies of the diet consumed to *Y*_m_, showed adequate accuracy at the farm level (root MSPE = 9.1%, CCC = 0.75, *R* ^2^ = .67). Our results thus suggest a new *Y*_m_ model and future challenges for estimating Zebu beef cattle production in tropical developing countries.

## 1 Introduction

With an estimated 999 million tons of carbon dioxide equivalent per annum, methane emissions from the enteric fermentation of beef cattle are a major human‐induced greenhouse gas emission (Opio et al., 2013). Enteric methane emissions represent a loss in the range of 2%–12% of the gross energy (GE) intake; that is, MJ methane energy loss per 100 MJ GE consumed by cattle, directly reducing the energy use efficiency of the diet consumed (Johnson & Johnson, 1995).

Currently, enteric methane emissions for cattle globally are estimated from energy requirements using the enteric methane conversion factor (*Y*_m_, % of GE intake), according to the IPCC (2006) standard. Practically, the *Y*_m_ default model of “*Y*_m_ = 6.5% ± 1.0% of GE intake” of IPCC (2006) (tier 2 level) is used worldwide to upscale national estimations and obtain accurate cattle population and related activity data. Average daily GE intake (MJ/day) and *Y*_m_ are ordinarily used to estimate methane emission factors. For nonfeedlot cattle (fed concentrate diet <90% of total intake), the upper bounds of the default model are recommended for diets with poorer digestibility and energy values (IPCC, 2006). Because the *Y*_m_ default model was developed from a dataset based on *Bos taurus* fed temperate feedstuffs, research that adopts country‐ or region‐specific *Y*_m_ models is also significant for reducing possible errors in the estimates of *Y*_m_ for different livestock and feed combinations (Lassey, 2007).

In addition to emissions at the national level, those at the farm level are also significant for applying methane mitigation strategies that may increase feed energy deposition in animals (Hristov et al., 2013). *Y*_m_ values at the farm level show extremely high variability (Johnson & Johnson, 1995; Lassey, 2007). Indeed, models that describe this circumstance at the farm level are too complex to be used in national inventories at low tier levels. Overall, extant models for estimating enteric methane emissions can be classified into two principal groups: empirical (statistical) or dynamic mechanistic models (Kebreab, Johnson, Archibeque, Pape, & Wirth, 2008). In terms of the former, independent variables such as animal and diet as well as energy utilization efficiency have been selected to develop empirical *Y*_m_ models (Blaxter & Clapperton, 1965; IPCC, 2006, Jaurena et al., 2015). Regarding the latter, a Danish‐specific *Y*_m_ model has been developed using a mathematical description of ruminal fermentation biochemistry (Nielsen et al., 2011).

One challenge is the lack of data available to predict *Y*_m_ for Zebu and Zebu crossbred beef cattle in tropical countries. This is a particular problem given that stocks of Zebu (*Bos indicus*) beef cattle in developing countries in tropical regions now account for more than half of the global beef cattle population (FAO, 2015). Both Kurihara, Magner, Hunter, and McCrabb (1999) and our previous studies (Chaokaur, Nishida, Phaowphaisal, & Sommart, 2015; Chuntrakort et al., 2014; Tangjitwattanachai, Phaowphaisal, Otsuka, & Sommart, 2015) have consistently found the *Y*_m_ value of Zebu beef cattle production in tropical regions to be much higher than those estimated by IPCC (2006). As the diets fed to these Zebu beef cattle typically consist of poor‐quality crop residues and by‐products compared with those fed to *B. taurus* (Kearl, 1982; NRC, 2000, WTSR, 2010), extant *Y*_m_ models may be inaccurate for the Zebu beef population.

Based on this gap in the body of knowledge on this topic, this meta‐analysis aimed to develop new and evaluate existing regional diet‐specific empirical *Y*_m_ models for Zebu beef cattle production in tropical regions at the national level and the farm level from on‐farm accessible data.

## 2 Materials and Methods

### 2.1 Dataset construction

A dataset was constructed from 18 energy balance or feeding experiments conducted from 1999 to 2015 (total 53 observations (*n*) as the feeding treatment means; from peer‐reviewed papers, proceedings, theses, and unpublished results from our research station) of Zebu and Zebu crossbred beef cattle fed low‐quality crop residues and by‐products in tropical regions (Canesin et al., 2014; Chaokaur, Nishida, & Sommart, 2010; Chaokaur et al., 2015; Chuntrakort et al., 2014; Hayashi et al., 2010 [unpublished results]; Kaewpila, Suzuki, & Sommart, 2015; Kennedy & Charmley, 2012; Khuamankgorn, Namsele, Angthong, & Martosoth, 2009; Kongphitee, Udchachon, Otsuka, & Sommart, 2010; Kongphitee et al., 2015 [unpublished results]; Kurihara et al., 1999; Moonmat, Otsuka, Udchachon, & Sommart, 2009; Nitipot, Pattarajinda, & Sommart, 2010; Phromloungsri, Hayashi, Otsuka, Udchachon, & Sommart, 2012; Sitthiwong, 2010; Suzuki et al., 2008; Tangjitwattanachai et al., 2015; Tomkins, McGinn, Turner, & Charmley, 2011). Diets that contained feed additive reagents for mitigating *Y*_m_ such as monensin were not included in the dataset, while starving animals and animals fed good‐quality forage and legumes or lipids supplements were also excluded. Enteric methane emissions were measured using an indirect respiration calorimeter (head hood) or a sulfur hexafluoride tracer technique. GE intake was measured by multiplying the GE content of the diet (determined using a bomb calorimeter) by dry matter intake (collected by total collection or a maker technique). Some previous studies have not reported nutritive values such as chemical composition, energy content, and feed digestibility, which are necessary as predictors for models in this meta‐analysis. Therefore, we investigated this missing information using animal feed information guidelines (Feedipedia, 2015, NRC, 2000, WTSR, 2010) including ether extract, neutral detergent fiber, and acid detergent fiber via the mean value of the feedstuffs. The procedures for determining the feed fractions are as follows (Mertens, 1997; Owens, Sapienza, & Hassen, 2010):

NFC=1,000−(Ash+CP+EE+NDF),

TDN=0.81×CP+2.23×EE+0.39×NDF+0.92×NFC.

Further, the models for predicting feed fractions were as follows (Rittenhouse, Streeter, & Clanton, 1971):

$$\text{DMD}=\left(\frac{\text{DE}/\text{GE}}{1.02\;-\;0.54}\right)\times 10,$$

$$\text{OMD}=\left(\frac{\text{DE}/\text{GE}}{1.07\;+\;8.13}\right)\times 10,$$

where NFC, nonfiber carbohydrates (g/kg DM); Ash expressed as g/kg DM; CP, crude protein (g/kg DM); EE, ether extract (g/kg DM); NDF, neutral detergent fiber (g/kg DM); TDN, total digestible nutrients (g/kg); DMD, dry matter digestibility (g/kg); DE, digestible energy (MJ/kg DM); GE, gross energy (MJ g/kg DM); and OMD, organic matter digestibility (g/kg). Note that the model inputs from some of these predicted parameters can further create additional errors beyond the model formulation. The summary statistics of the dataset are shown in Table 1.

**Table 1:** Summary statistics of the Zebu beef cattle dataset used to develop and evaluate the models (*n *=* *53)

Item	Mean	*SD*	Minimum	Maximum
Beef cattle				
Age (month)	23	10	12	48
Body weight (kg)	277	80	113	432
Diet composition (g/kg dry matter)				
Roughage proportion	526	287	220	1,000
Organic matter	911	25	840	962
Crude protein	106	33	40	213
Ether extract	36	16	10	78
Neutral detergent fiber	507	142	293	756
Acid detergent fiber	296	93	162	472
Nonfiber carbohydrates	260	123	53	543
Energy content (MJ/kg dry matter)				
Gross energy	17.6	1.4	15.0	19.9
Digestible energy	11.9	1.7	8.3	14.8
Metabolizable energy	10.1	1.7	6.7	12.9
Digestibility (g/kg)				
Dry matter digestibility	643	70	464	746
Organic matter digestibility	677	72	508	790
Total digestible nutrients	604	86	454	737
Feeding level				
Dry matter intake (kg/day)	4.6	1.5	2.2	7.7
Dry matter intake (% body weight)	1.7	0.3	1.2	2.2
Metabolizable energy intake^a^	1.4	0.3	1.0	2.2
Enteric methane emission				
Methane emission (g/day)	123	53	38	311
*Y*_m_ (% gross energy intake)	8.2	1.7	4.8	13.7

a Expressed as multiple time of maintenance requirement (0.48 MJ ME/kg BW^0.75^, WTSR, 2010).

### 2.2 Extant *Y*_m_ model selection

The extant *Y*_m_ models from the published works (Blaxter & Clapperton, 1965; FAO, 2010; IPCC, 2006, Jaurena et al., 2015, Patra, 2013; Ramin & Huhtanen, 2013; Yan, Agnew, Gordon, & Porter, 2000) presented in Table 2 were selected to predict the *Y*_m_ of the beef cattle dataset and to guide the model development. The model selection criteria were based on the model's possible use at the national or farm level as well as the existence of independent variables in the dataset. At the national level, the tier 2 *Y*_m_ default models of the IPCC (2006) are used worldwide (Kebreab et al., 2008). These *Y*_m_ estimates are a rough guide based on the beef farm practices in most developed and developing countries (IPCC, 2006). A default model (*Y*_m_ = 6.5% ± 1.0% of GE intake, namely model A) was selected to emphasize the IPCC's recommendation for cattle fed low‐quality crop residues and by‐products in developing countries. At the farm level, regression models are typically used to estimate the *Y*_m_ values related to the complex variable(s) of the animal and its diet. Seven regression models (namely model B, C, D, E, F, G, and H) were thus selected to increase the levels of the complexity variable(s).

**Table 2:** Extant models selected to predict *Y*_m_ values

Model category	Model	Relationship^a^	Description
National level			
IPCC (2006)	Model A	*Y*_m_ = 6.5 ± 1.0	These IPCC guidelines for the tier 2 level are used to upscale the measurements of national and global inventories. The model is developed from a database including dairy cows in New Zealand, dairy heifers and steers in the United States, and beef cows in France (i.e., animals grazing in temperate pastures). It is the currently suggested emissions inventory method for the enteric fermentation of cattle population categories fed low‐quality crop residues and by‐products in developing countries
Farm level			
Patra (2013)	Model B	*Y*_m_ = 7.10 − 0.0192 × EE	This model is developed from a database including dairy and beef cattle fed a wide range of dietary composition in unspecified locations
Yan et al. (2000)	Model C	*Y*_m_ = [0.0522 + 0.0694 × ADFI/DMI] × 100	This model is developed from a database including dairy cows and steers offered grass silage‐based diets in Northern Ireland
FAO (2010)	Model D	*Y*_m_ = 9.75 − 0.005 × DMD	This model has been previously used to predict methane emissions from dairy cattle production in Sweden and Nigeria. No information on the database is available
Jaurena et al. (2015)	Model E	*Y*_m_ = Intercept alternatives^b^ − 0.243 × DMI + 0.0059 × NDF + 0.0057 × DMD	This model is developed from a database including beef cattle fed a wide range of dietary composition in unspecified locations
Ramin and Huhtanen (2013)	Model F	*Y*_m_ = [–0.60 − 0.70 × DMIbw + 0.076 × OMDm − 0.130 × EE + 0.046 × NDF + 0.044 × NFC]/10	This model is developed from a database including dairy and beef cattle and sheep fed a wide range of dietary composition in unspecified locations
Yan et al. (2000)	Model G	*Y*_m_ = [0.0877 − 0.0078 × (MEIm − 1.00)] × 100	See description of model C
Blaxter and Clapperton (1965)	Model H	*Y*_m_ = 1.30 + 11.2 × DE/GE − [2.37 − 5.00 × DE/GE] × MEIm	This model is developed from a database including cattle and sheep fed roughages or mixed diets in the United Kingdom

a *Y*_m_, methane conversion factor (% of GEI); DE, digestible energy (MJ/kg DM); GE, gross energy (MJ/kg DM); GEI, GE intake (MJ/day); MEIm, metabolizable energy intake as multiple time of maintenance requirement (0.48 MJ ME/kg BW^0.75^, WTSR, 2010); ADFI, acid detergent fiber intake (kg/day); DMI, dry matter intake (kg/day); DMD = dry matter digestibility(g/kg); DMIbw, dry matter intake (g/kg body weight); OMDm, organic matter digestibility (OMD) determined at a maintenance level of feeding (g/kg, OMDm = OMD (g/kg) + 1.83 × [DMIbw − 10]); NDF, neutral detergent fiber (g/kg DM); EE, ether extract (g/kg DM); NFC, nonfiber carbohydrates (g/kg DM).

b All the intercept alternatives were used: fresh forage with level of concentrate less than 35% (of dry matter intake), and between 35% and 65% = 2.0, and 4.1 (respectively), conserved forages with level of concentrate less than 35%, between 35% and 65%, and more than 65% = 3.1, 2.3, and 1.5 (respectively), straw with level of concentrate less than 35%, between 35% and 65%, and more than 65% = 5.1, 4.4, and 1.0 (respectively).

#### 2.2.1 Model development for the national level

This model (Table 3, namely model I) was simulated according to the tier 2 level of IPCC (2006), which developed a *Y*_m_ model based on the quotient of mean methane energy emissions to mean GE intake across the measured herd, while the conversion from methane energy to flux in mass units was 55.56 MJ/kg (Lassey, 2007). Thus, the calculation was

(1) $$Y_{m}=\frac{\left(\sum_{i=1}^{n}{\text{CH}}_{4i}\right)/n}{\left(\sum_{i=1}^{n}{\text{GEI}}_{i}\right)/n}\times 100,$$

where *Y*_m_, methane conversion factor (% of GE intake); CH_4*i*_, the *i*th observed methane energy emissions (MJ/day); GEI_*i*_, the *i*th observed GE intake (MJ/day); and *n*, number of observations. Based on the IPCC's recommendation, the *Y*_m_ value for the national level can be estimated although the uncertainty in the mean (i.e., choices regarding what to include or exclude), which originally relate to the digestibility and energy values of the diet. The uncertainty in the mean was calculated as ±1.96 multiples of the standard error (IPCC, 2006).

**Table 3:** List of models developed to predict the *Y*_m_ values

Model category	Model^a^	*p*‐Value	*R* ^2^ b	RMSPE %^c^	Largest VIF^d^
National level (*n *=* *53)					
Model I	*Y*_m_ = 8.4 ± 0.4	–	–	–	–
Farm level (*n *=* *36)					
Model J (dietary level)	*Y*_m_ = 14.12_(*SE* = 1.55, *p* < .01) _− 0.073_(*SE* = 0.018, *p* < .01)_ × EE − 0.006_(*SE* = 0.002, *p* < .05)_ × NDF	<.01	.34	17.4	1.29
Model K (intake level)	*Y*_m_ = 7.70_(*SE* = 0.69, *p* < .01) _− 8.33_(*SE* = 3.40, *p* < .05)_ × EEI + 3.74_(*SE* = 0.89, *p* < .01)_ × CPI	<.01	.38	16.9	1.04
Model L (digestibility level)	*Y*_m_ = − 0.24 _(*SE* = 2.53, *p* = .92)_ + 0.013_(*SE* = 0.004, *p* < .01)_ × OMD	<.01	.25	18.5	–
Model M (integrated dietary, intake and digestibility level)	*Y*_m_ = 8.65_(*SE* = 1.05, *p* < .01)_ − 0.034_(*SE* = 0.015, *p* < .05)_ × EE − 1.41_(*SE* = 0.38, *p* < .01)_ × DMI + 2.57_(*SE* = 0.54, *p* < .01)_ × DOMI	<.01	.54	14.5	8.03
Model N (energy level)	*Y*_m_ = 37.70_(*SE* = 5.00, *p* < .01)_ + 19.71_(*SE* = 2.69, *p* < .01)_ × DE/GE − 50.70_(*SE* = 6.55, *p* < .01)_ × ME/DE	<.01	.71	12.0	1.24

a *Y*_m_, methane (CH_4_) conversion factor (% of GE intake); EE, ether extract (g/kg DM); NDF, neutral detergent fiber (g/kg DM); EEI, ether extract intake (g/day); CPI, crude protein intake (g/day); OMD, organic matter digestibility (g/kg); DOMI, digestible organic matter intake (kg/day); GE, gross energy (MJ/kg DM); DE, digestible energy (MJ/kg DM); ME, metabolizable energy (MJ/kg DM).

b *R* ^2^, coefficient of determination.

c RMSPE, root‐mean‐square prediction error (% of the mean of observed *Y*_m_).

d VIF, variance inflation factors (>10 indicates existing of collinearities among the independent variables).

#### 2.2.2 Model development for the farm level

This investigation was carried out using a subsampling dataset (*n *=* *36, termed the two‐thirds dataset) from the total dataset (*n *=* *53). The models were developed using a multiple linear regression analysis, which relates the independent variable(s) to *Y*_m_. This investigation was conducted in a sequential manner to increase model complexity at each level and thus increase the model's predictive power, which is based on complex information (IPCC, 2006, Moraes, Strathe, Fadel, Casper, & Kebreab, 2014). According to the dataset availability and extent models (Table 2), five complexity levels were performed, namely dietary, intake, digestibility, integrated dietary, intake and digestibility, and energy levels (Table 3). All variables were computed under the selected most probable model at these levels of complexity. Specifically, the regression analysis for model complexity at each level was analyzed using the REG procedure (stepwise and collinearity diagnostics) of the SAS statistical software version 6.12 (SAS Institute Inc. Cary, NC, USA). The statistical model was

(2) $$Y_{m}\;=\;\beta_{0}\;+\;\beta_{1}X_{1}\;+\;\beta_{2}X_{2}\;+\;,\ldots ,\;+\;\beta_{n}X_{n}\;+\;\varepsilon ,$$

where *Y*_m_ = methane conversion factor (% of GE intake); β_0_ = intercept, β_1_, β_2_, …, β_*n*_ = slopes, *X*_1_, *X*_2_, …, *X*_*n*_ = independent variables, and ɛ = error.

#### 2.2.3 Cross‐evaluation

Three statistical parameters, namely the coefficient of determination (*R* ^2^), root‐mean‐square prediction error (RMSPE), and variance inflation factors (VIFs), were undertaken to evaluate the developed models against the observed *Y*_m_ to assess model performance. The predicted *Y*_m_ dataset for each model was developed using the model regressor. Model I, developed for the national level, was excluded as it did not exactly mimic the real regression system.

The *R* ^2^ (stepwise) and VIFs (collinearity diagnostics) were obtained during the model development process previously described via the REG procedure of the SAS. This *R* ^2^ was used as an index of the goodness of fit of the *Y*_m_ models, determining the proportion of variance in the observed *Y*_m_ explained by the model (Nakagawa & Schielzeth, 2013). Thus, *R* ^2^ of 1 indicates that the regression line perfectly fits the data, while an *R* ^2^ of 0 indicates that the line does not fit the data at all. The VIFs measure the inflation in the variances of the parameter estimates due to collinearities that exist among the independent variables (Belsley, Kuh, & Welsch, 1980). The largest VIF was used as the formal criterion for deciding if it is larger than 10 (i.e., sufficient to affect the predicted values; Moraes et al., 2014).

The RMSPE was calculated as

(3) $$\text{RMSPE}=\sqrt{\frac{\sum_{i=1}^{n}{\left(O_{i}-P_{i}\right)}^{2}}{n}}\times 100/\overset{¯}{O},$$

where *O*_*i*_ = *i*th observed *Y*_m_ value, *P*_*i*_ = *i*th predicted *Y*_m_ value, *n *= observation number, and $\overset{¯}{O}$ = mean observed *Y*_m_ value. The RMSPE was used as an index to describe the predictive accuracy of every developed model (Tedeschi, 2006). RMSPE values are expressed as a percentage of the observed *Y*_m_, and range from 0 to positive infinity. An RMSPE value equal to 0 indicates a perfect score in the predictive accuracy model.

### 2.3 Comparison of the extant and developed *Y*_m_ models using on‐farm accessible data

This comparison aimed to evaluate the performance of the extant and developed models in predicting *Y*_m_ using on‐farm accessible data (the one‐third dataset, *n *=* *17). The predicted *Y*_m_ values were constructed as a dataset by adding the independent variable(s) into the *Y*_m_ models. Models A and I for the national level were again used. The predicted *Y*_m_ values for models A and I were generated around their mean value using their specific uncertainty value, namely 6.5% ± 1.0% GE intake and 8.4% ± 0.4% GE intake, respectively. The mean was presumed to be the lower bounds if the diet had a greater DE/ME value (due to the negative relationship between the energy use efficiency and *Y*_m_ values), and thus, the upper bounds were used on the opposite side.

Three parameters were used as model evaluation tools, namely the mean square prediction error (MSPE; Tedeschi, 2006), the concordance correlation coefficient (CCC; Lin,^1989^), and observed versus predicted values (Kebreab et al., 2008). These statistical analyses are widely used to assess biological models (Ellis, Bannink, France, Kebreab, & Dijkstra, 2010; Jaurena et al., 2015; Tedeschi, 2006).

The MSPE analysis was divided into RMSPE (3) and total MSPE. Total MSPE was decomposed to compile the sources of variation in the MSPE (Bibby & Toutenburg, 1977), consisting of errors in central tendency (ECT), errors due to regression (ER), and errors due to disturbances (ED). These four statistical parameters were calculated as

(4) Total MSPE=ECT+ER+ED,

(5) $$\text{ECT}\;=\;{\left(\overset{¯}{O}\;-\;\overset{¯}{P}\right)}^{2},$$

(6) $$\text{ER}\;=\;{\left(S_{P}\;-\;r\;\times \;S_{O}\right)}^{2},$$

(7) $$\text{ED}\;=\;\left(1\;-\;r^{2}\right)\;\times \;S_{O}^{2},$$

where $\frac{}{O}$ = mean observed *Y*_m_ value, $\overset{¯}{P}$ = mean predicted *Y*_m_ value, *S*_P_ = standard deviation of the predicted *Y*_m_ value, *r *= Pearson correlation coefficient, and *S*_O_ = standard deviation of the observed *Y*_m_ value. ECT, ER, and ED were expressed as a percentage of total MSPE.

The CCC and derivative statistics (Lin, 1989) consisting of *r*, bias correction factor (*C*_b_), and the location shift (μ) were calculated as

(8) $$\text{CCC}\;=\;r\;\times \;C_{b},$$

(9) $$C_{b}=\frac{2}{\left(S_{O}/S_{P}\right)\;+\;\left(1/\left(S_{O}/S_{P}\right)\right)\;+\;\mu^{2}},$$

(10) $$\mu =\frac{\overset{¯}{O}\text{-}\overset{¯}{P}}{\sqrt{S_{O}\;\times \;S_{P}}},$$

where the notations are as above. The CCC evaluates a model's predictive accuracy and precision at the same time (the degree to which the pairs of observed and predicted *Y*_m_ values fall on the unity line). A CCC value equal to 1 indicates perfect agreement between the two variables (Tedeschi, 2006). This *r* measures precision (deviation of the observed from the predicted *Y*_m_ values line) and *C*_b_ (range = 0___1, perfect score = 1) measures accuracy (how far the predicted *Y*_m_ values line deviates from the unity line) (Ellis et al., 2010). Moreover, μ (range = negative___positive infinities, perfect score = 0) is a measure of the location shift relative to the scale (the degree of the residual of means relative to the root of the product of two standard deviations). A positive μ value indicates underprediction, while a negative one indicates overprediction (Kebreab et al., 2008).

The observed versus predicted plots were analyzed using the method described by Ellis et al. (2010). Briefly, the slope was determined by regressing the observed *Y*_m_ values (independent variable) against the predicted *Y*_m_ values (dependent variable) using the REG procedure. This response aims to test the significance of the slope against 0, which assesses the existence of linear relationship between the observed and predicted values.

## 3 Results

### 3.1 Dataset description

The dataset for this meta‐analysis, including beef cattle characteristics (age and body weight), diet composition, digestibility, feeding level, and enteric methane emissions, is shown in Table 1. The GE content of diets ranged from 15.0 to 19.9 MJ/kg DM and averaged 17.6 MJ/kg DM. Enteric methane emissions were ~120 g head^−1^ day^−1^ (range 38–311 g head^−1^ day^−1^). The observed *Y*_m_ value ranged from 4.8% to 13.7% of GE intake.

### 3.2 Development of the models

**Figure 1:**
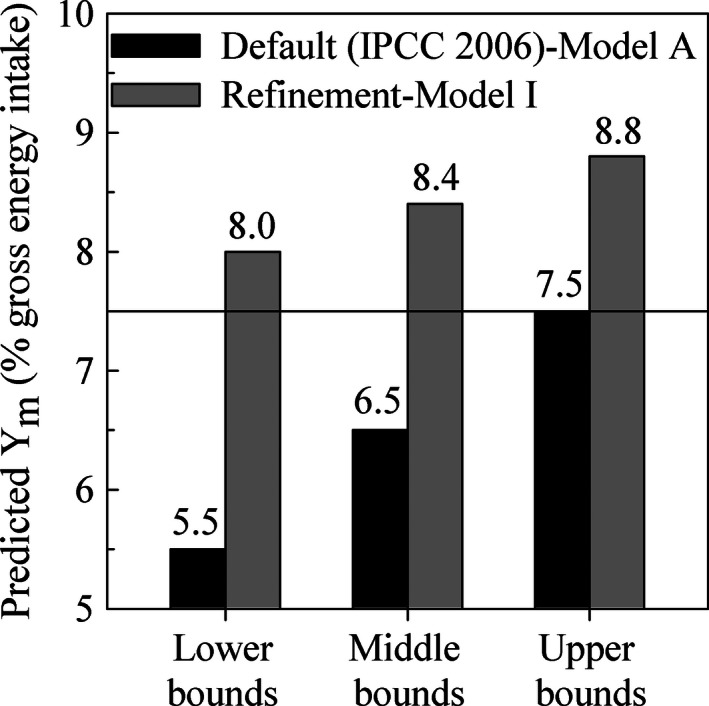
Methane conversion factor (*Y*_m_) of Zebu beef cattle fed low‐quality crop residues and by‐products in tropical regions as compared with the IPCC default values. The referent line (*Y*_m_ = 7.5%) represented the limitation of the IPCC default model

The *Y*_m_ models developed and categorized using the levels of predictive possibility discussed herein are listed in Table 3. For the national level, the model simulated according to the IPCC yielded model I as the refinement (*Y*_m_ = 8.4% ± 0.4% of GE intake) to the IPCC default model (*Y*_m_ = 6.5% ± 1.0% GE intake). Based on a model comparison (Figure 1), the IPCC default model underestimated the *Y*_m_ values of the Zebu beef cattle fed low‐quality crop residues and by‐products in tropical regions by up to 45.5%, 29.3%, and 17.3% at the lower, middle, and upper bounds, respectively.

For the farm level, the regression analysis yielded models J, K, L, M, and N (*p* < .01; Table 3) that represented the dietary (ether extract and neutral detergent fiber contents of the diet), intake (ether extract and crude protein intakes), digestibility (organic matter digestibility), integrated dietary, intake and digestibility (ether extract content, dry matter intake, and digestible organic matter intake), and energy (DE/GE and ME/DE) levels, respectively. The independent variable(s) in models J, K, L, and M did not fit well to predicted *Y*_m_ values because they had too low *R* ^2^ (.25___.54), and relatively moderate RMSPE values (14.5%___18.5%). The multiple variables in model N had a moderate *R* ^2^ of .71 and a low RMSPE of 12.0%, and thus presumably had a moderate fit among all models. As it showed the largest VIF of 1.24, the clarity of the collinearity between the DE/GE and ME/DE variables was demonstrated.

### 3.3 Comparison of the extant and developed models using on‐farm accessible data

The MSPE analysis (Table 4) indicated that model N was the best performing model here (RMSPE = 9.1%, of which 99.7% of this error came from the disturbance). The CCC analysis also selected model N as that having the highest precision and accuracy (CCC = 0.75, *r *=* *.77, *C*_b_ = 0.97) among the evaluated models. The positive and low μ value for model N (μ = 0.03) indicated a slightly underpredicted *Y*_m_ value.

**Table 4:** Mean predicted *Y*_m_ values and analysis of the MSPE and CCC of the extant and developed *Y*_m_ models (using the one‐third dataset, *n *=* *17)

Model category	Mean of predicted*Y*_m_ (±*SE*)^a^	MSPE analysis^b^				CCC analysis^c^			
		RMSPE%	ECT%	ER%	ED%	CCC	*r*	*C*_b_	μ
National level									
Model A	6.56 (±0.135)	23.1	64.6	0.6	34.8	0.18	.52	0.33	1.78
Model I	8.42 (±0.054)	15.1	7.0	14.2	78.8	0.16	.53	0.31	−0.62
Farm level									
Model B	6.42 (±0.086)	24.8	67.0	3.2	29.8	0.11	.54	0.20	2.43
Model C	7.34 (±0.185)	20.1	20.4	16.7	62.9	0.04	.06	0.70	0.75
Model D	6.58 (±0.088)	25.1	53.9	4.8	41.3	0.02	.07	0.23	2.18
Model E	7.80 (±0.414)	22.2	2.5	51.3	46.2	0.27	.28	0.95	0.20
Model F	7.01 (±0.130)	18.6	48.6	2.0	49.4	0.23	.57	0.45	1.28
Model G	8.48 (±0.053)	16.2	8.4	0.2	91.4	0.06	.21	0.30	−0.73
Model H	10.14 (±0.422)	36.0	47.9	32.2	19.9	0.02	.05	0.51	−0.14
Model J	8.32 (±0.289)	14.1	3.6	14.4	82.0	0.54	.55	0.98	−0.19
Model K	8.08 (±0.148)	12.2	<0.1	4.6	95.4	0.48	.63	0.76	0.01
Model L	8.08 (±0.221)	18.6	<0.1	28.1	71.9	0.06	.06	0.93	0.01
Model M	8.08 (±0.230)	11.3	<0.1	0.6	99.4	0.62	.66	0.95	0.01
Model N	8.06 (±0.254)	9.1	0.2	0.1	99.7	0.75	.77	0.97	0.03

a *Y*_m_, methane conversion factor (% of GE intake); mean of the observed *Y*_m_ is 8.09 (*SE* = ±0.321).

b MSPE, mean square prediction error; RMSPE, root‐mean‐square prediction error (% of the observed mean); ECT, errors in central tendency (% of total MSPE); ER, errors due to regression (% of total MSPE); ED, errors due to disturbances (% of total MSPE).

c CCC, concordance correlation coefficient; *r*, Pearson correlation coefficient; *C*_b_, bias correction factor; μ, location shift.

**Figure 2:**
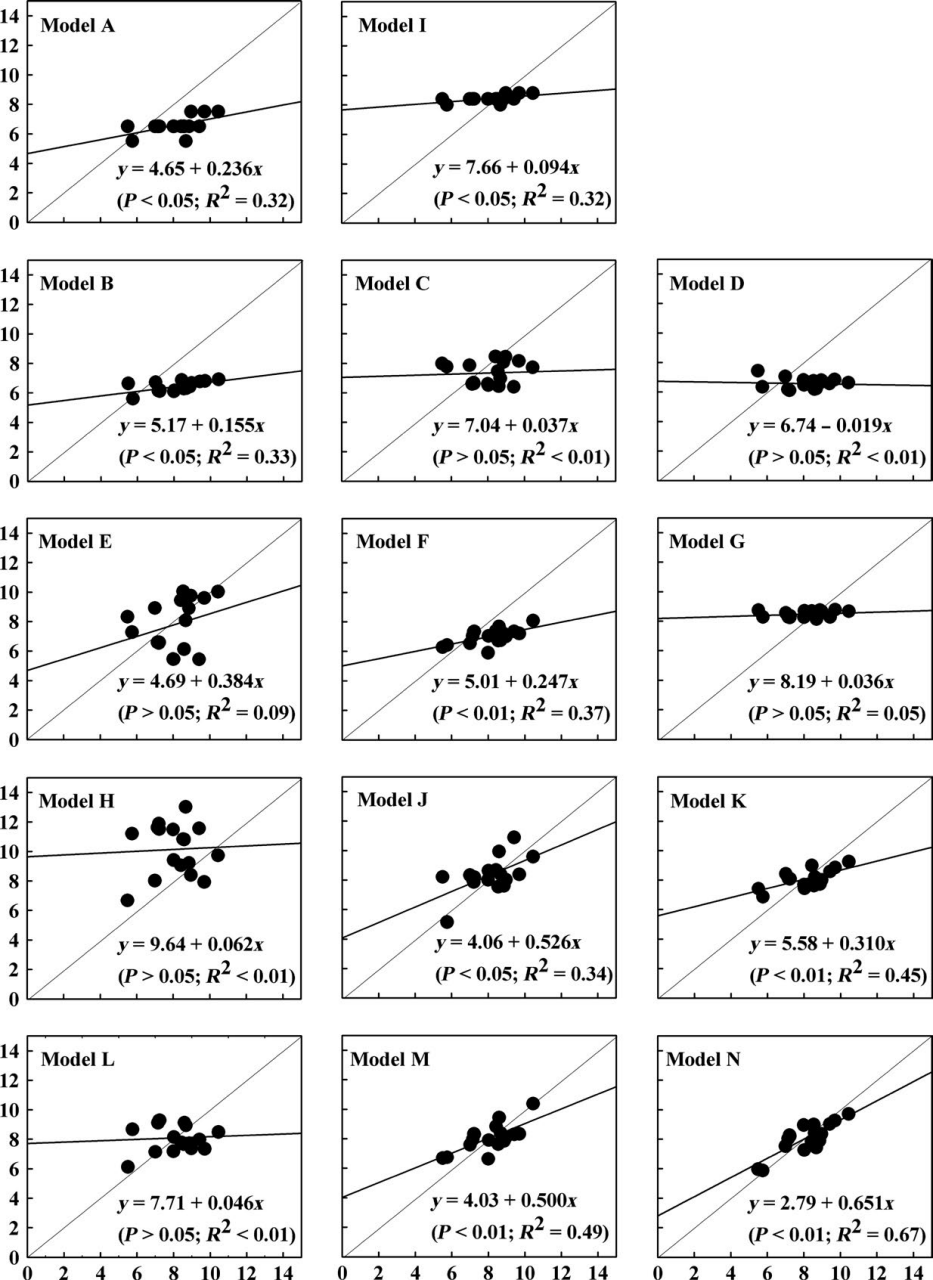
Predicted (*y*‐axis) versus observed (*x*‐axis) *Y*_m_ values of the extant and developed models (using the one‐third dataset, *n *=* *17)

Once again, the analysis of the observed versus predicted values plots (Figure 2) indicated that only model N had moderate predictive power (*R* ^2^ = .67). For most of the models here, although the statistical significance of the slope was reached (*p* < .05 or <.01), predictive power was very low considering an *R* ^2^ less than .50.

## 4 Discussion

### 4.1 Perspectives of the dataset

Because of the importance of *Y*_m_ in determining the accuracy of enteric methane emissions for national and global inventories (IPCC, 2006), we analyzed a range of *Y*_m_ models of the associated beef cattle production system. The present dataset (Table 1) is different from that used to develop the IPCC (2006) *Y*_m_ default model, by means of geographic areas of data, and especially the existence of cattle breeds and feed resources from tropical areas highlighted here. Our dataset covered a wide range beef cattle fed low‐quality crop residues and by‐products production systems in tropical regions (from growing to finishing). Zebu beef cattle in Thailand such as native Thai cattle have a low mature body weight of ~450 kg for females and ~550 kg for males, while Brahman cattle and Zebu beef cattle crossed with *B. taurus* can show a higher mature body weight (Marcondes, Tedeschi, Valadares Filho, & Gionbelli, 2013; Ogino et al., 2016). Nellore beef cattle, wide spread in Brazil and India, are also a small breed size, with a mature body weight of ~530 kg according to Marcondes et al. (2013). The diet compositions and nutritive values such as crude protein (40–213 g/kg DM), total digestible nutrients (454–737 g/kg), and ME content (6.7–12.9 MJ/kg DM) showed several available feeding systems for tropical developing countries. The mean enteric methane emission rate in our records (~120 g methane head^−1^ day^−1^) could result from frame size and voluntary feed intake of cattle (Smith, Lyons, Wagner, & Elliott, 2015).

The range of *Y*_m_ values of 4.8%–13.7% of GE intake in this study agreed with that in Johnson and Johnson (1995). The many attempts to estimate *Y*_m_ variability emphasize the difficulty because of the number of factors related to *Y*_m_ (Blaxter & Clapperton, 1965; Hill, McSweeney, Wright, Bishop‐Hurley, & Kalantar‐Zadeh, 2016). What is certain is the positive relationship between plant fiber digestion and high acetic acid production sides as well as between plant fiber digestion and high methanogenesis yields in the rumen (IPCC, 2006, Jaurena et al., 2015). Roughage sources can change *Y*_m_ because of the fiber compositions (Jaurena et al., 2015; Kennedy & Charmley, 2012). While dietary lipid is also undeniably a strong single indicator of *Y*_m_, the change is not constant (e.g., when sources of the lipid are different; Patra, 2013). Further, diets fed as single feed at varying levels of feeding also deduce *Y*_m_ given the stimulated rates of passage or undigested feed in the rumen (Blaxter & Clapperton, 1965; Chaokaur et al., 2015). Using these factors as the single or multiple variables of a *Y*_m_ model are typical, while rough estimates often fail to capture *Y*_m_ for a variety of reasons including extrapolation (application of the model beyond the domain for which model predictions are known to be valid; IPCC, 2006, Bannink, van Schijndel, & Dijkstra, 2011). Studies have suggested that the application of models is positively associated with the degree of representativeness between a model's dataset and a real farm (Ellis et al., 2010, IPCC, 2006). Ideally, models which relate the diet particles and chemical component rates of passage and digestion in each enteric compartment at varying intake levels and the resulting hydrogen balance, volatile fatty acids, and microbial yields should generate *Y*_m_ values that are reliable to direct measurements from cattle (IPCC, 2006). Thus, more representative and complex models can result in fairly different *Y*_m_ accuracies (i.e., the degree of simulation of real systems).

Many attempts also promoted the methane mitigation through consideration of model's prediction characteristics. Recently, Jaurena et al. (2015) demonstrated that the categories of cattle (beef or dairy) and roughage (fresh forage, conserved forage, straw) are the primary factors affecting *Y*_m_ values. Conventionally, it has been known that *Y*_m_ values reduce as ME intake (Blaxter & Clapperton, 1965; Chaokaur et al., 2015), feed quality (starch content), energy content (IPCC, 2006; Johnson & Johnson, 1995; Kurihara et al., 1999), and fat content rise (Chuntrakort et al., 2014; Patra, 2013). Our previous studies showed that an increase in feeding level not only reduces *Y*_m_ values, but also improves beef productivity; thus, reducing the intensity of enteric methane is a strategic feeding management approach (Chaokaur et al., 2015; Tangjitwattanachai et al., 2015).

### 4.2 Predicting *Y*_m_ values at the national level for Zebu beef cattle in tropical regions

The predicted *Y*_m_ values are used in a complex algorithm standardized by IPCC (2006). If inventory compliers are chosen at the tier 2 level, the aim is to control errors of less than 20% around the mean of the enteric methane emission inventory of a country. IPCC (2006) suggested that a 10% error in a variable will result in methane errors ranging up to 20% depending on the circumstances. Our result (Figure 1) showed room to improve *Y*_m_ predictions for Zebu beef cattle fed low‐quality crop residues and by‐products in tropical regions. Compared with the refinement (model I), the default underestimated by up to 29.3% for the reference animal and diets (*Y*_m_ = 6.5% vs. 8.4% GE intake). This finding confirmed the *Y*_m_ degrees are different among different livestock and feed combinations (IPCC, 2006). However, the available data are sparse during the *Y*_m_ default models. The 0.4% GE intake of uncertainty for predicted *Y*_m_ was lower than the 1.0% reported under tier 2 because of the sample size effect (i.e., a larger sample size reduces its standard error). The sample size in the present dataset was larger than that of Lassey (2007) used to develop the IPCC's *Y*_m_ default model, that is, *n *=* *53 versus *n *=* *14. Traditionally, the uncertainty of the refinement (model I) could also be replaced as 1.0% GE intake (IPCC, 2006). Beyond the scope of our study, as the available data are limited, such an improvement still needs cattle fed on tropical pastures as well a tier 3 model that includes a dynamic and mechanistic model of fermentation biochemistry in the enteric to calculate enteric methane emission inventories, instead of a tier 2 one (Bannink et al., 2011). The *Y*_m_ tabulation for the cattle fed blooming grasses, legumes, and high‐quality crop residues should be related to the IPCC's data because there is evidence in Brazil and Australia that the *Y*_m_ response to this diet is rather similar given the overall range of uncertainty (Kennedy & Charmley, 2012; Pedreira et al., 2013; Tomkins et al., 2015). Additionally, a main reason for this difference is the degree to which *Y*_m_ depends on feed quality (Jaurena et al., 2015; Kurihara et al., 1999; Lassey, 2007).

### 4.3 Predicting the *Y*_m_ values at the farm level for Zebu beef cattle in tropical regions

Predicting the *Y*_m_ values at the farm level is a different task compared with tier 2. Indeed, describing the *Y*_m_ trends from the direct measurements in cattle is challenging for a variety of reasons such as the importance of data on methane mitigation throughout the assessment of the carbon footprint values (Ogino et al., 2016). According to Kebreab et al. (2008), predicted values equal observed values in a perfect model. Thus, the best model should have a low RMSPE, high CCC, and high *R* ^2^ (observed vs. predicted). Some researchers have shown that regression models may also be capable of describing the changes in *Y*_m_ values considering the effects of dietary changes (Blaxter & Clapperton, 1965; Jaurena et al., 2015; Patra, 2013; Ramin & Huhtanen, 2013; Yan et al., 2000). In particular, as the regression approach statistically relates the factors of animal and diet to *Y*_m_ output, it was thus effective to refine to the IPCC (2006) default *Y*_m_ model when predicting at the farm level because the latter is designed to enumerate national‐level emissions (Crosson et al., 2011). Our results (Table 4, Figure 2) showed that model N had adequate predictive performance among the examined models. The results recognized that a large scatter of *Y*_m_ values that represent on‐farm data needs complexity for generating estimates, including DE/GE and ME/DE (energy use efficiencies of the diet consumed). The positive relationship between DE/GE and the *Y*_m_ value agrees with the findings of Blaxter and Clapperton (1965). In the IPCC (2006) guidelines, DE/GE is recommended as an important factor for controlling variations in *Y*_m_, although it is excluded in the tier 2 model. For model N, the ME/DE appeared to be an additional implement beyond to other traditions. Indeed, the *Y*_m_ value was sensitive to variation in ME/DE because methane is an energy loss that is represented in DE to ME content. Model N's assessment of an enteric methane inventory relies on the beef cattle herds and feedstock being well characterized. In tropical regions of developing countries, some farmers impose changes in beef herd composition and feeding regime to improve beef productivity. These considerations challenge the enteric methane inventory method (Lassey, 2007).

For the case of extent models, the lack of data representativeness of the cattle used in this analysis could be a major source of error. This kind of model error typically calls for extrapolation, which is associated with a lack of correspondence between the circumstances associated with the available data and those associated with the predictions (IPCC, 2006). In this case, the *Y*_m_ data of the extent models may be available for situations in which high diet quality is stimulating at high voluntary intake load but not for situations involving the intake limited changes due to low diet quality (Table 1, dry matter intake varied from 1.2% to 2.2% body weight). Thus, the variables are only partly relevant to the desired *Y*_m_ estimate. Another possible error is the measurement error, which may be random as a result of missing information (feed characteristics, includes ether extract, fibers, and digestibilities) from external sources. Overall, the results imply that the further prediction of *Y*_m_ should focus on representing the effects of traditional variables such feed characteristics and intakes.

In conclusion, this meta‐analysis highlighted the advantages of some developed *Y*_m_ models for Zebu beef cattle fed low‐quality crop residues and by‐products in tropical developing countries. The dataset reported the importance of Zebu beef cattle, diet composition, feeding level, enteric methane emission rate (~120 g head^−1^ day^−1^), and *Y*_m_ model (8.4% ± 0.4% of GE intake) for the national level regarding the IPCC's tier 2 level application. We further showed that the IPCC default model (*Y*_m_ = 6.5% ± 1% of GE intake) underestimates the *Y*_m_ value by 1.9% of GE intake. At the farm level, seven of the extant models examined herein were inadequate for describing changes in the *Y*_m_ value (RMSPE = 16.2%–36.0%, CCC = 0.02–0.27, *R* ^2^ = <.01–.37). Finally, although these findings contribute to our understanding of Zebu beef cattle populations in tropical regions and offer better model applications for estimating their presented *Y*_m_ values, the lack of information obtained from feedlot and grazing herds is a limitation of this study. Thus, the scopes for further research should be to develop the *Y*_m_ models using feedlot and grazing datasets to provide more implications in estimating enteric methane emissions of Zebu cattle.

## Conflict of Interest

None declared.
